# Avascular necrosis of humeral head in an elderly patient with tuberculosis: a case report

**DOI:** 10.1186/1752-1947-2-361

**Published:** 2008-12-04

**Authors:** Renu Agarwal, Ruchika Gupta, Sompal Singh, Kusum Gupta, Madhur Kudesia

**Affiliations:** 1 Department of Pathology, Hindu Rao Hospital, Malka Ganj, Delhi-110007, India; 2 Department of Pathology, All India Institute of Medical Sciences, Ansari Nagar, New Delhi-110029, India

## Abstract

**Introduction:**

Osteonecrosis (avascular necrosis) is known to be caused by high-dose corticosteroid therapy, alcoholism and rarely by infections. However, a tubercular etiology of this condition is very rare. A review of the literature yielded only a few cases of polyarticular tuberculosis with osteonecrosis in immunosuppressed individuals. No case of monoarticular tubercular osteonecrosis diagnosed by aspiration cytology was found. Since tuberculosis is a curable disease, an early and accurate diagnosis is essential.

**Case presentation:**

A 60-year-old Indian man presented with diffuse swelling and pain in the left shoulder for the previous 6 months. A computed tomography scan of the left shoulder revealed crescentic lucency in the humeral head, suggestive of osteonecrosis. Fine needle aspiration cytology smears from the swelling showed features of an acute suppurative lesion. Stain for acid-fast bacillus was positive and thus, a final clinico-pathological diagnosis of osteonecrosis of humeral head with tubercular etiology was rendered. The patient was initiated on anti-tuberculous therapy with symptomatic improvement in his condition.

**Conclusion:**

Osteonecrosis, a debilitating disease, may rarely occur due to tuberculosis, especially in endemic areas. Fine needle aspiration cytology is an effective and inexpensive modality for an early diagnosis of the tubercular etiology of osteonecrosis.

## Introduction

Osteonecrosis, also known as avascular necrosis (AVN), occurs in people with risk factors such as high-dose corticosteroid therapy, excessive alcohol intake, injury, malignancy, systemic lupus erythematosus, and hematologic disorders such as sickle cell disease [1]. Among infectious causes, Human Immunodeficiency Virus (HIV) and meningococcemia have been reported to cause AVN [2,3]. However, AVN in association with tuberculosis has been reported in only a few cases [4,5]. In one case, described by Cheung et al. in 1995, polyarticular tuberculosis with AVN was identified in a HIV positive patient [4]. No case of monoarticular tuberculosis associated with AVN has been reported in the available literature.

Our case depicts a rare association of monoarticular tuberculosis with AVN in an immunocompetent patient.

## Case presentation

A 60-year-old man, Indian by origin, presented with swelling and pain in the left shoulder of 6 months duration. There was associated anorexia and loss of weight. However, there was no history of preceding trauma, corticosteroid therapy or significant medical or surgical treatment. He was a non-alcoholic and non-smoker.

**Figure 1:**
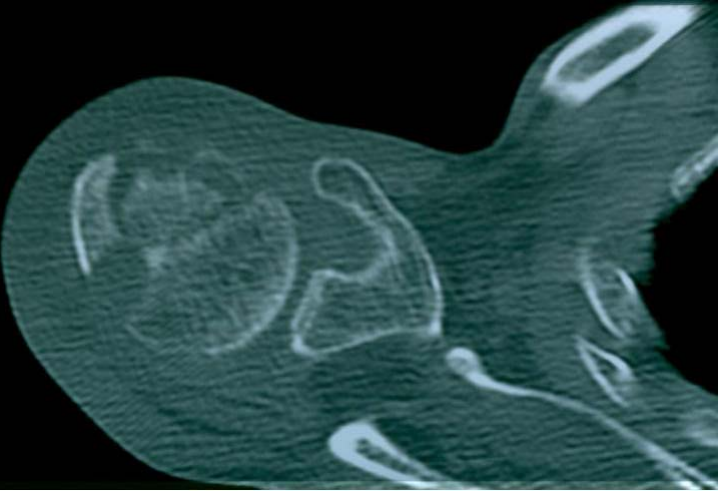
Computed tomography scan at the level of the upper humerus showing crescentic lucency as evidence of osteonecrosis.

On local examination, a diffuse, soft and tender swelling, 4 × 4 cm, was seen at the left shoulder. There was mild restriction of movement of the left shoulder. The overlying skin was warm and erythematous. Hematological investigations revealed peripheral blood lymphocytosis and increased erythrocyte sedimentation rate (ESR) (35 mm in the first hour). Mantoux test using 5 tuberculin units (TU) of purified protein derivative (PPD) showed significant induration after 72 hours (13 mm). Serological tests for HIV, rheumatoid factor and anti-nuclear antibodies (ANA) were negative. Chest X-ray did not show any evidence of active/healed pulmonary tuberculosis. Radiographs of the left shoulder joint did not reveal any bony abnormality. Computed tomography (CT) scan of the left shoulder showed a crescentic lucency in the humeral head with associated soft tissue swelling, consistent with a diagnosis of osteonecrosis (Figure 1). The patient was referred for fine needle aspiration (FNA) cytology of the soft tissue swelling to assist in etiological diagnosis.

**Figure 2:**
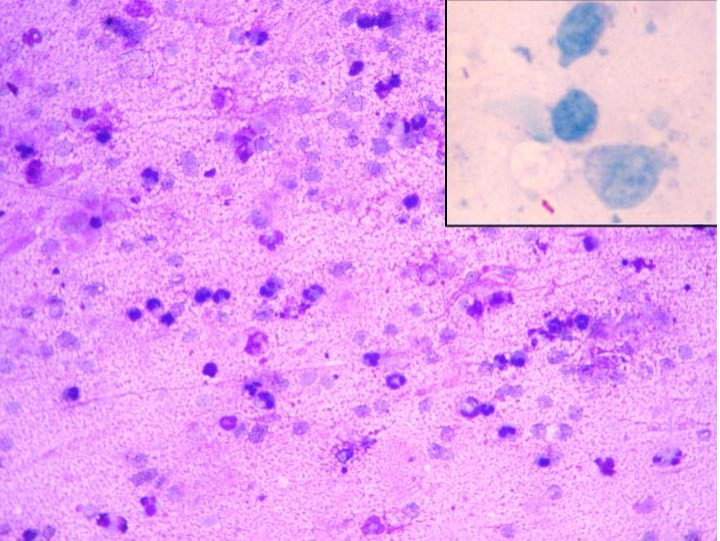
**Fine needle aspiration smear showing many viable and degenerating neutrophils in a thin necrotic background. **Inset shows an acid-fast bacillus (Giemsa Stain ×200, Inset: Ziehl Neelsen Stain ×400).

FNA yielded a purulent aspirate, smears which showed an acute suppurative lesion with intact and degenerated neutrophils in a proteinaceous background along with a few lymphocytes and histiocytes (Figure 2). No epithelioid cell granulomas were noted. Ziehl Neelsen staining showed occasional acid-fast bacilli (Figure 2, inset). A diagnosis of tubercular etiology of osteonecrosis was rendered. The patient was put on antitubercular therapy, after which the pain and swelling reduced markedly.

## Discussion

Osteonecrosis, also known as avascular necrosis (AVN), aseptic necrosis or ischemic necrosis, results from temporary or permanent loss of blood supply to a part of bone. As a result of the loss of blood supply, the bone may ultimately collapse [1].

Numerous risk factors have been associated with AVN including corticosteroid therapy, alcohol intake and bony injury. Other associations include systemic malignancy, lupus erythematosus, sickle cell disease, Gaucher's disease, Caissons disease, gout, vasculitis, osteoarthritis, osteoporosis, radiation therapy, chemotherapy and organ transplantation, particularly renal transplant [1]. A rare causal association with infections such as HIV and meningococcemia (with disseminated intravascular coagulation) has been reported [2,3]. However, a large number of cases do not have any obvious etiologic factor and are reported as idiopathic [1].

Various imaging techniques have been used for diagnosis of AVN. Plain X-ray has a low sensitivity and shows bone damage only in later stages of disease. CT scan is better than X-rays, however, its sensitivity compared to magnetic resonance imaging (MRI) is still low, especially in early stages of the disease [1]. MRI is currently the accepted standard for noninvasive diagnosis. The classical MRI appearance of AVN is that of a segmental area of low signal density in the subchondral bone on T1-weighted images [1]. In our patient, a plain radiograph did not reveal AVN, which could be picked up on CT scan. MRI could not be performed due to lack of facilities.

The goal of treatment of AVN is to improve the use of the affected joint, stop further damage to the bone and ensure bone and joint survival. The underlying cause of AVN has to be ascertained and eliminated if possible. Surgical intervention, including arthroscopic debridement, core decompression, vascularized bone grafting and bone reconstruction, is advocated when symptoms are persistent and signs of collapse are evident [1].

Tuberculosis, caused by *Mycobacterium tuberculosis*, is a common infectious disease in the developing countries and is re-emerging in developed nations due to the human immunodeficiency virus (HIV) pandemic [6]. Osteoarticular tuberculosis results from hematogenous dissemination of *Mycobacterium tuberculosis *from a primary infected visceral focus to the skeletal system [7]. Our present case adds to the myriad of radiological presentations of osteoarticular tuberculosis, i.e. avascular necrosis. AVN with tuberculosis as an etiological cause of osteonecrosis has only been mentioned in rare case reports. Two cases of AVN of femoral capital epiphysis following intertrochanteric tubercular osteomyelitis have been reported [5]. There is a recent report describing AVN in a HIV positive patient with polyarticular tuberculosis [4]. To the best of our knowledge, the present case is the first report documenting an association of monoarticular tuberculosis with AVN in an immunocompetent patient, where the etiology of osteonecrosis was confirmed on aspiration cytology. In this patient, FNA showed an acute suppurative lesion with a few acid-fast bacilli. This case underlines the utility of Ziehl-Neelsen stain to diagnose tubercular etiology in cases with radiological diagnosis of AVN, especially when the patient is a resident of an endemic zone.

## Conclusion

This case report emphasizes that tuberculosis should be retained as one of the important differential diagnoses in cases of osteonecrosis, especially in endemic areas, after other more common etiological disorders have been excluded. Aspiration cytology offers a rapid, yet inexpensive method for diagnosis leading to appropriate therapy being initiated.

## Abbreviations

ANA: anti-nuclear antibody; AVN: avascular necrosis; CT: computed tomography; ESR: erythrocyte sedimentation rate; FNA: fine needle aspiration; HIV: Human Immunodeficiency Virus; MRI: magnetic resonance imaging; PPD: purified protein derivative; TU: tuberculin units.

## Consent

Written informed consent was obtained from the patient for publication of this case report and any accompanying images. A copy of the written consent is available for review by the Editor-in-Chief of this journal.

## Competing interests

The authors declare that they have no competing interests.

## Authors' contributions

RA performed the fine needle aspiration cytology and wrote the case outline. RG was a major contributor in writing the manuscript and revising it. SS assisted in reviewing the slides, provded images and helped in final drafting of the manuscript. KG assisted in the literature review and writing of the manuscript. MK interpreted the fine needle aspiration cytology and critically reviewed the manuscript for its intellectual content. All authors have read and approved the final manuscript to be published. All authors have participated sufficiently to take public responsibility of the content of the manuscript.
